# Bilateral orbital plasmacytomas as first sign of extramedullary progression post CAR-T therapy: case report and literature review

**DOI:** 10.3389/fonc.2023.1217714

**Published:** 2023-08-10

**Authors:** Javier Nogués-Castell, Silvia Feu-Basilio, Óscar Felguera García, Carlos Fernández de Larrea, Aina Oliver-Caldés, Olga Balagué Ponz, Jessica Matas Fassi

**Affiliations:** ^1^ Institut Clínic d’Oftalmologia, Hospital Clínic de Barcelona, Universitat de Barcelona, Barcelona, Spain; ^2^ Institut D’ Investigacions Biomèdiques August Pi i Sunyer (IDIBAPS), Fundació Clínic per a la Recerca Biomèdica (FCRB), Universitat de Barcelona, Barcelona, Spain; ^3^ Amyloidosis and Myeloma Unit, Department of Hematology, Hospital Clínic de Barcelona, Universitat de Barcelona, Barcelona, Spain; ^4^ Centre de Diagnòstic Biomèdic, Hospital Clínic de Barcelona, Universitat de Barcelona, Barcelona, Spain

**Keywords:** plasma cells leukemia, chimeric antigen receptor therapy, CAR-T, orbital plasmacytoma, orbital multiple myeloma

## Abstract

**Background:**

Plasma cell leukemia (PCL) is an aggressive and rare form of plasma cell dyscrasia characterized by peripheral blood expression, poor prognosis, and high relapse rates. Extramedullary plasmacytomas are common in this entity and can affect various organs and soft tissues. Chimeric antigen receptor–T-cell (CAR-T) therapy is a novel immunotherapy for hematological malignancies with promising results. However, it is not indicated for PCL, and experience in this condition is limited. This case is a rare presentation of bilateral orbital plasmacytomas after CAR-T therapy in a patient with PCL history.

**Case presentation:**

We present the case of a 51-year-old female patient with a history of previous primary PCL treated with CAR-T therapy achieving complete response and without evidence of systemic progression. Six months after the treatment, she developed subacute proptosis and ptosis on the left eye.

An orbital CT scan was performed and showed an orbital tumor in both eyes. A surgical biopsy with histological examination revealed plasma cells, consistent with a plasmacytoma. PET-CT and MRI confirmed the presence of tumors in both orbits. The patient was treated with dexamethasone and chemotherapy along with palliative radiation therapy to the left orbit which had a good response.

**Conclusion:**

Orbital involvement in multiple myeloma and PCL is rare, with plasmacytomas being more common in other parts of the body. In this report, we present a case of a patient with PCL history, treated with multiple therapeutic lines including CAR-T therapy, who presented bilateral orbital plasmacytomas as the first sign of extramedullary progression after the treatment. This case should be considered by specialist to be aware that the orbits are a possible location of extramedullary progression.

## 1 Introduction

Plasma cell leukemia (PCL) is a rare form of plasma cell dyscrasia and the most aggressive form of the human monoclonal gammopathies. Previous studies have reported an incidence rate of PCL between 2% and 4% of patients with multiple myeloma (MM) (1–4). Recent European studies from the HAEMCARE project found a crude incidence of PCL in the European population of 0.4 per million, accounting for approximately 0.5% of MM cases (5).

PCL diagnosis criteria have been recently redefined from 20% plasma cells in peripheral blood leukocytes or an absolute plasma cell count of ≥2 × 10^9^/L to the presence of more than 5% plasma cells in peripheral blood leukocytes or an absolute plasma cell count of ≥0.5 × 10^9^/L (6). Studies from the International Myeloma Working Group (IMWG) have shown that the presence of peripheral blood cells leads to more aggressive MM, and the presence of ≥5% circulating plasma cells in patients with MM has an adverse prognostic value similar to the patients with higher percentage rates (7, 8). Thus, the incidence of PCL has shown an increase between 0.7% and 2.5%, being the latter from a multicenter Catalan series (7, 8).

The clinical presentation of PCL is usually aggressive and develops from a fast and furious tumor burden, with deep cytopenia and a high rate of extramedullary involvement. The most common locations of extramedullary involvement in PCL are the liver, spleen, lymph nodes, lungs, central nervous system (CNS) or soft tissue plasmacytomas (3). Unlike MM, PCL rarely presents with osteolysis (3). Given the very high rate of extramedullary disease, the IMWG has suggested that fluorodeoxyglucose (FDG)–PET/CT should be considered in the diagnosis, evaluation, and monitoring of PCL (9). Survival of patients with PCL is short due to resistance to therapy, despite receiving multiple lines (6). Treatment of PCL typically includes induction combination regimens with immunomodulatory drugs and proteasome inhibitors followed by autologous hematopoietic stem cell transplantation (ASCT) and post-ASCT multidrug maintenance therapy with novel agents. Allogeneic stem cell transplantation (alloSCT) has also been also performed in these patients to improve survival rates (10).

Plasmacytomas are soft tissue neoplasms formed by a monoclonal plasma cell and may be associated with MM or PCL (11). Orbital plasmacytomas are extremely rare, accounting for only 1% of orbital tumors (12, 13). They may occur in association with plasma cell dyscrasias or isolated, although 50% of isolated plasmacytomas progress to MM within a year. Orbital Plasmacytomas may be the first manifestation of a systemic disease and the first sign of relapse (12–15).

The most common presenting sign of orbital plasmacytomas is proptosis. Reduced visual acuity, oedema, and diplopia are also commonly reported. In MM, they tend to be unilateral and have a slow progression. The most commonly affected quadrant is the superior-temporal (14). Plasmacytomas in MM have usually good response to radiotherapy (4, 14).

## 2 Case presentation

**Figure 1:**
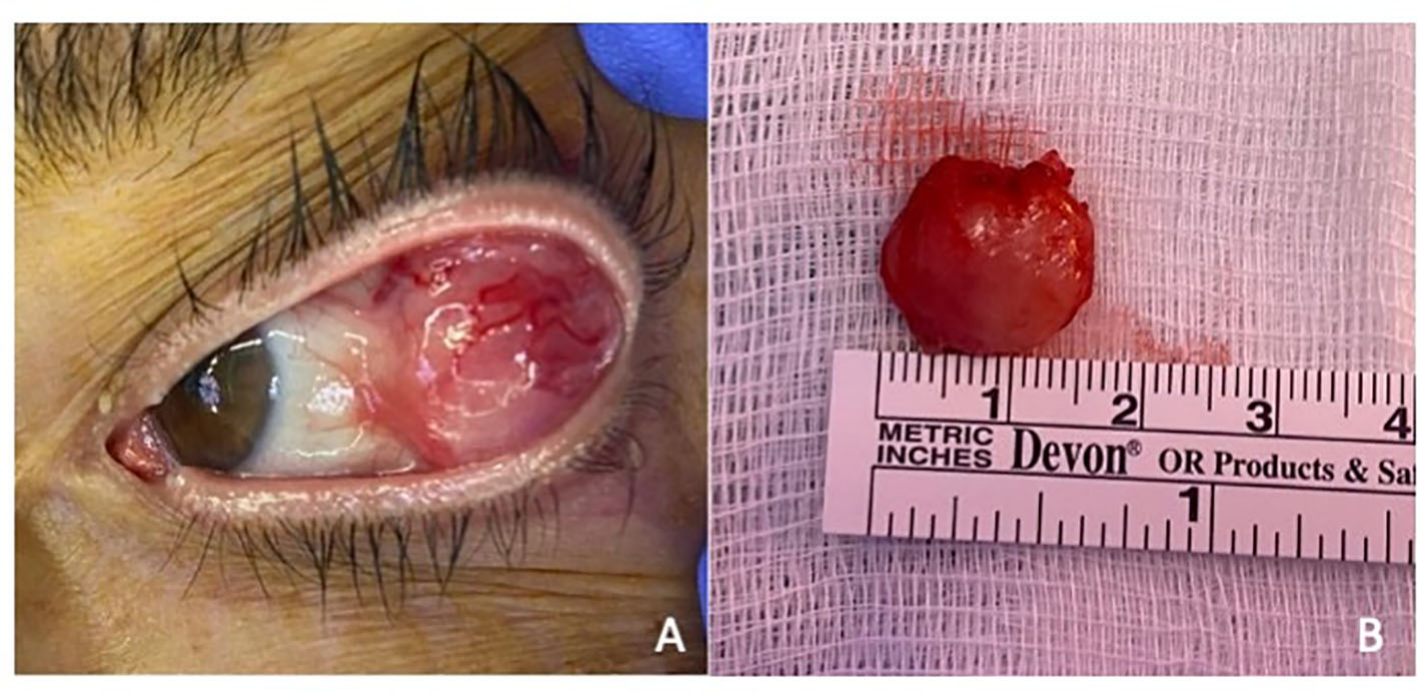
**(A)** Orbital tumor presentation. Orbital mass in the left eye presenting subconjunctival extension and no evidence of adherence to the globe or eyelids. **(B)** Surgical biopsy of the left orbital mass. Macroscopic view of the excised red colored mass measuring 16 mm × 14 mm × 12 mm with an elastic consistency.

We present a 51-year-old woman who consulted the ophthalmology emergency department with proptosis on the left eye, oedema, and superior palpebral induration of one week duration (**Figure 1A**). Medical relevant history included a breast cancer in 2012 treated surgically and with tamoxifen until 2015, and she had a prothrombin 20210A mutation and was diagnosed in 2015 of PCL for which she received multiple therapy lines, as detailed in **Table 1**. Ophthalmologically, she had a history of dry eye due to graft-versus-host disease (GVHD).

The patient was diagnosed of primary PCL in a different center in August 2015. The initial presentation was of a severe pneumonia with bad evolution due to cytopenia. The laboratory test made at the moment of presentation manifested leucocytosis with a high number of circulating atypic plasma cells (49%). The patient also showed proteinuria, high blood levels of B2-microglobulins, and presence of light chains in serum. Examination of the marrow bone showed more than 69% plasma cell invasion. A PET-CT scan was run without signs of extramedullary disease. The plasma cells karyotype showed structural and numeric alterations including 1q trisomy and chromosome 13 monosomy, conclusive of a bad prognosis. The patient was diagnosed of IgG-lambda isotype primary PCL with no signs of extramedullary involvement. She received several lines of treatment, including alloSCT, as described in **Table 1**. Thus, 6 months prior to the ophthalmology emergency room consultation, she was treated with ARI0002h, an academic chimeric antigen receptor (CAR)–T-cell (CAR-T) therapy against BCMA (TNFRSF17) as a compassionate use.

Before CAR-T administration, patient had extramedullary affection in the form the hepatic hilum plasmacytomas with severe hepatic compromise, proteinuria, and high IgG levels in serum. However, marrow bone examination before treatment only showed 1% of plasma cells. After CAR-T administration, complete remission was achieved, with good clinical response and no evidence of disease in peripheral blood, as well as bone marrow examination and radiological stability in PET-CT imaging.

**Figure 2:**
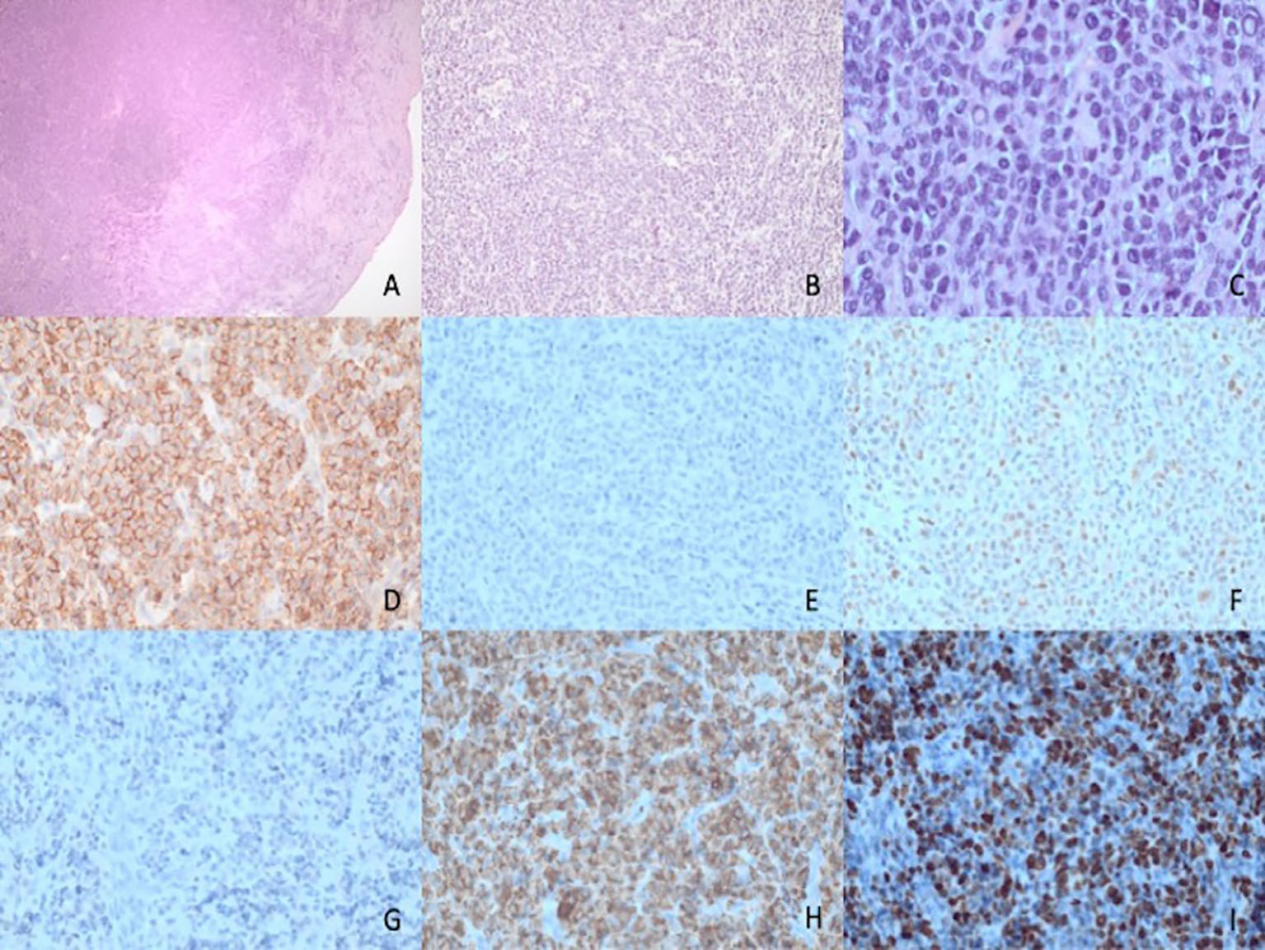
Histopathological features of plasma cell neoplasm with pleomorphic features. **(A–C)** Histopathological features showing a dense subepithelial infiltrate **(A)** of a medium-sized cells with diffuse distribution. **(B)** At higher magnification, **(C)** the tumor cells show plasma cell differentiation with Dutcher bodies but a higher level of pleomorphism than expected for a mature plasma cell proliferation. **(D)** Positive staining for CD138 and negative staining for CD20 **(E)** with partial positivity for cyclin D1 **(F)**. Staining for kappa **(G)** and lambda **(H)** shows lambda light-chain restriction. In addition, staining for KI67 **(I)** shows a proliferation index of around 70%, higher than expected for a mature plasma cell proliferation.

Ophthalmic examination revealed a visual acuity of 80/80 in both eyes. Extraocular and intraocular movements were intact, and she denied diplopia or pain. Orbital palpation revealed a mass in the temporal superior quadrant. Intraocular pressure was within normal limits in both eyes. Orbital CT scan revealed an orbital mass in the superior-temporal quadrant. Because of the presentation and history of breast cancer and PCL, the first suspected diagnosis was of a malignant tumor. A surgical biopsy of the lesion was performed through superior-temporal conjunctival incision (**Figure 1B**). The pathology analysis revealed a plasma cell dyscrasia with light-chain lambda restriction and a high proliferative index. All findings were consistent with a diagnosis of plasmacytoma, suggestive of extramedullary progression (**Figure 2**).

**Figure 3:**
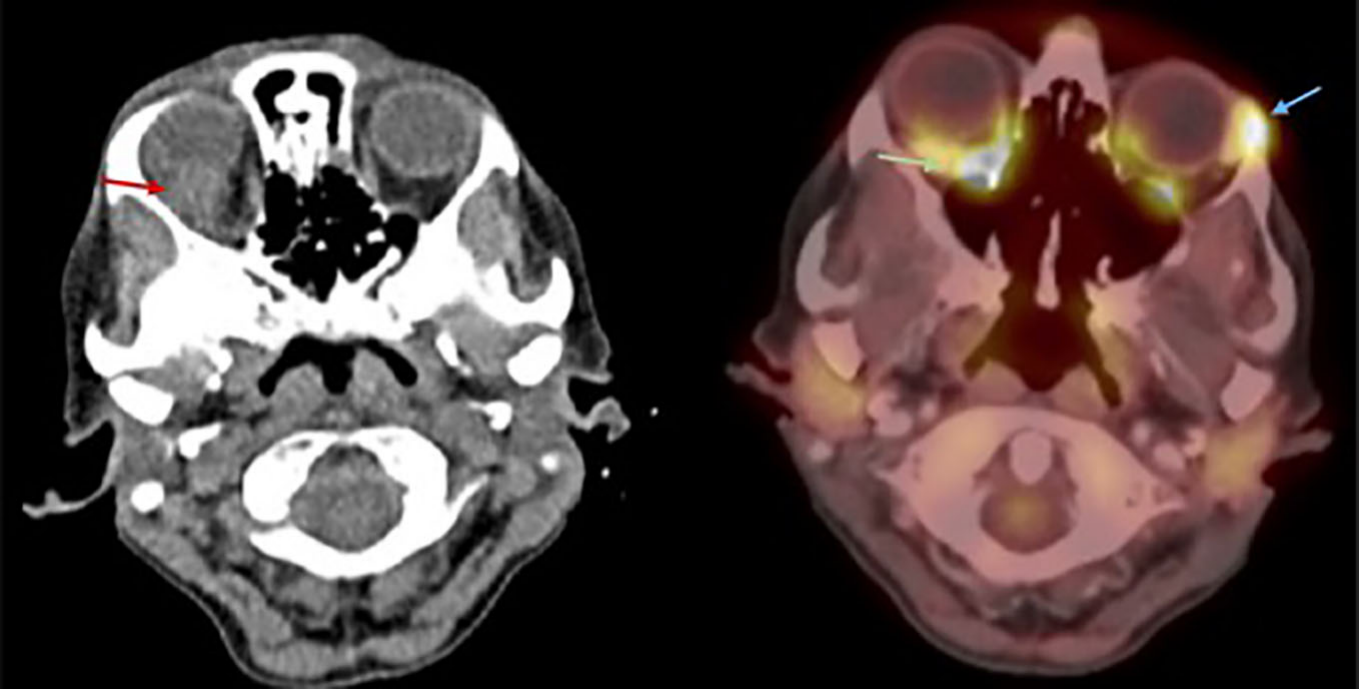
Axial MRI and PET-CT. Red arrow: Right orbital mass in the superior-temporal quadrant. Green arrow: Right orbital mass with increased FDG uptake. Blue arrow: Surgical bed after left orbital biopsy with high FDG uptake, possibly related to postsurgical inflammation.

Body PET-CT scan and orbital MRI were performed after the pathology results to exclude other tumor lesions, evidencing an orbital mass in the right orbit without any other signs of activity in other organs (**Figure 3**). Blood and urine analysis revealed the presence of monoclonal lambda chains (65.00 U/mg/L) and an elevated b2 microglobuline level. Plasma cell count in peripheral blood samples showed no evidence of plasma cells in the smear. Marrow bone analysis performed after the biopsy results showed 1% plasma cells. Hematologists initiated a palliative treatment due to plasmacytoma rapid progression with steroids and chemotherapy. Radiation therapy of 20 Gy in 10 fractions was administered to the right orbit with a good radiological and clinical response.

Two weeks after the surgery on the left eye, the orbital and ocular examinations revealed residual hyperemia and fibrosis at the surgical site. Four months later, there was a clinical plasmacytoma recurrence of the left orbit with proptosis and a palpable extraocular mass in the superior- temporal quadrant. A local radiotherapy at 16 Gy in 4 fractions was performed with both clinical and radiological resolution. Blood tests were repeated periodically showing a progressive increase in lambda chain count of more than a hundred times in the following 3 months (1,590.00 U/mg/L). CT scans were run periodically after the relapse, showing signs of extramedullary progression in the mesenterial affecting the intrahepatic biliary duct and the right cardiophrenic fat with pleural involvement. Six months after the orbital plasmacytoma biopsy, the patient suffered from ascites. The ascitic cytology analysis showed plasma cell infiltration. Because of extramedullary progression and sepsis, palliative measures were provided.

## 3 Treatment timeline

**Table 1:** Lines of treatment in the patient.

Line	Treatment	Date	Treatment-related complications
First line	VTD-PACE × 4 (bortezomib, thalidomide, and dexamethasone–cisplatin, doxorubicin, cyclophosphamide, and etoposide) with a very good partial response (VGPR); allogeneic stem cell transplantation	2016	Chronic graft-versus-host disease (GVHD)
Second line	KRD (carfilzomib, lenalidomide, and dexamethasone) and local radiotherapy to extramedullary plasmacytomas in liver and bones	2017	GVHD reactivation
Third line	Daratumumab monotherapy	2017	-
Fourth line	VCD (bortezomib, cyclophosphamide, and dexamethasone)	2017	Peripheral neuropathy
Fifth line	PoCyDex (pomalidomide, cyclophosphamide, and dexamethasone) × 25 cycles every 28 days: complete remission	2018	-
Sixth line	CAR-T against BCMA (ARI0002h) with compassionate use; previous lymphodepletion regimen with cyclophosphamide and fludarabine	2021	–
Eighth line	Local radiotherapy + cyclophosphamide, C carfilzomib, and prednisone	February 2022	–
Ninth line	Cyclophosphamide + local radiotherapy	May 2022	–

## 4 Discussion and conclusions

Plasmacytomas can be classified as medullary, occurring only within the bone, or extramedullary, occurring in soft tissues. The latter may be paraskeletal (in contact with a bone) or due to hematogenous dissemination.

In 2009, Burkat et al. reviewed the existent literature and found that half of all documented cases of orbital plasmacytomas occurred in patients who had already been diagnosed with MM (14). However, orbital involvement in PCL, as in the reported case, has been described in very few cases (16).

Our patient presented with proptosis and oedema on the left eye, with a mass in the superior-temporal quadrant. According to the literature, this is the most common form of presentation of orbital plasmacytoma, with proptosis being a common finding and the superior-temporal quadrant being the most common location, due to a rich blood supply that may favor metastasis. Reduced vision, swelling, and ptosis have also been frequently reported. Rarely, ecchymosis, cellulitis, and necrobiotic xanthogranuloma may be seen (14).

Orbital symptoms in orbital MM are typically insidious, with an average of 5 months from symptom onset to presentation (14). Bone involvement is also characteristic of MM. In contrast, our case presented with rapid growth and no bone involvement. Such a different clinical presentation could be due to the aggressive presentation of PCL compared with the insidious presentation of MM. Previous immunotherapies, including alloSCT and CAR-T cells, may have also affected the type of presentation.

Although orbital plasmacytomas are usually unilateral, bilateral manifestations, as in our case, have been reported (16–18). Orbital plasmacytomas have been described as the first manifestation of systemic disease and also as the first sign of relapse, like in our case (12–15). Some organs may act as sanctuaries, where graft-versus-leukemia (GVL) effect and chemotherapy cannot penetrate sufficiently (17). The eyes, testes, and CNS may act as such, with a lack of GVL effect. The orbit, with its direct relationship to CNS, is thought to have the same effect. Reports of isolated orbital relapse after alloSCT suggest a similar shielding effect in other diseases such as non-Hodgkin lymphoma (19, 20). Bilateral involvement as a form of relapse in our patient and the previously described cases may be explained by this theory.

In addition, orbital imaging is not easy to assess. In our case, CT and body PET-CT scans were performed after CAR-T therapy periodically to exclude any type of early systemic relapse, and no signs of orbital affection were detected by imaging before orbital presentation, with the latest PET-CT scan being performed only 20 days before. PET-CT can be misinterpreted in this area, as normal PET uptake can be seen at the apex and along the length of the extraocular muscles, masking tumors (21). Furthermore, body CT and PET-CT scans usually do not include orbital and CNS cuts if not asked specifically. Thus, orbital presentation can become a delayed diagnosis, so it is important for the radiologist to be highly trained in orbital imaging.

New treatments such as CAR-T therapy are being tested for plasma cells discrasias. CAR-T therapy consists in using genetically engineered autologous T cells that are programmed to bind specific antigens on target cells. Promising results have been reported in the treatment of some lymphomas, leukemia, and MM, but there are still few data on the response on PCL as is not indicated (4, 9, 22, 23).

Allogeneic transplantation is now rarely used in MM, especially in the first-line setting (24). However, because of the lack of long-term disease control with the therapeutic strategies used in recent years for primary PCL, the use of this immunotherapy in the first line has been proposed as a potential option in the last decade (9). More recently, several publications have shown that allogeneic transplantation may not be very useful compared with autologous transplantation, even in such aggressive disease. A tandem approach may still be useful in this setting. The early incorporation of advanced targeted immunotherapies, such as CAR-T cells and bispecific antibodies, should be explored in early lines for this disease.

Between 2020 and 2022, several new CAR-T therapies have emerged for both newly diagnosed MM and relapsed or refractive MM, and two have been approved in European Union although without reimbursement in Spain (25). ARI2000H is a second-generation lentiviral autologous CAR-T targeting BCMA with a 4-1BB and signal transduction CD3 co-stimulatory domain and a humanized single-chain variable fragment. ARI2000H has shown potency *in vitro* and *in vivo* activity in preclinical studies and has demonstrated an excellent feasibility in a clinical trial with deep and durable responses and a promising safety profile (26). Compassionate use of ARI2000H is ongoing (26).

The efficacy of CAR-T therapy in extramedullary MM is still in under debate. Previous reports have shown that plasmacytomas may not respond to this therapies in patients with extramedullary disease (27).

This case is the first reported case of bilateral orbital plasmacytoma presentation in a patient with history of PCL after the use of any CAR-T. In the future, it would be of clinical interest to be aware of this orbital condition in patients treated with these novel therapies.

In conclusion, we present a case of bilateral orbital plasmacytoma as the first sign of myeloma relapse after CAR-T therapy. To our knowledge, it is the first case of PCL orbital involvement after this therapy described in the literature and one of the few described cases of bilateral orbital plasmacytomas in PCL; its rapid growth and lack of bone involvement differ from the orbital plasmacytomas seen in MM.

Bilateral orbital involvement of this unusual entity seems extremely rare, which might indicate an orbital predisposition. Therefore, the possibility of orbital affection should be known by specialist to detect it promptly. If, in the future, more cases of orbital affection are reported, then a comprehensive orbital examination in patients with this disease who have been treated with CAR-T therapy might have to be contemplated. This also highlights the importance of interdisciplinary collaboration in this setting.

## Patient perspective

Unfortunately, due to the PCL progression the patient was deceased before publishing this article.

## Ethics approval and consent to participate

Not applicable.

## Ethics Statement

Written informed consent was obtained from the patients/participants for the publication of any potentially identifiable images or data included in this case study.

## Author contributions

JNC participated in the case description, background research, and discussion. SFB, OFG and JMF reviewed the ophthalmological data and ensured the accuracy of the bibliography used. AOC and CFL reviewed the haematology data of the case and the final discussion. OBP provided the histopathological description. All authors read and approved the final version of the manuscript.
